# Draft genome and description of *Waterburya agarophytonicola* gen. nov. sp. nov. (Pleurocapsales, Cyanobacteria): a seaweed symbiont

**DOI:** 10.1007/s10482-021-01672-x

**Published:** 2021-10-21

**Authors:** Guido Bonthond, Sergei Shalygin, Till Bayer, Florian Weinberger

**Affiliations:** 1 Institute for Chemistry and Biology of the Marine Environment (ICBM), Carl von Ossietzky University Oldenburg, Schleusenstrasse 1, 26382 Wilhelmshaven, Germany; 2 GEOMAR Helmholtz Centre for Ocean Research Kiel, Düsternbrooker Weg 20, 24105 Kiel, Germany; 3 Plant and Environmental Sciences Department, New Mexico State University, 945 College Drive, Las Cruces, NM 88003 USA

**Keywords:** *Gracilaria vermiculophylla*, Cobalamin, Holobiont, Pleurocapsales, Symbiosis, Vitamin B_12_

## Abstract

This work introduces *Waterburya agarophytonicola* Bonthond and Shalygin gen. nov., sp. nov, a baeocyte producing cyanobacterium that was isolated from the rhodophyte *Agarophyton vermiculophyllum* (Ohmi) Gurgel et al., an invasive seaweed that has spread across the northern hemisphere. The new species genome reveals a diverse repertoire of chemotaxis and adhesion related genes, including genes coding for type IV pili assembly proteins and a high number of genes coding for filamentous hemagglutinin family (FHA) proteins. Among a genetic basis for the synthesis of siderophores, carotenoids and numerous vitamins, *W. agarophytonicola* is potentially capable of producing cobalamin (vitamin B_12_), for which *A*. *vermiculophyllum* is an auxotroph. With a taxonomic description of the genus and species and a draft genome, this study provides as a basis for future research, to uncover the nature of this geographically independent association between seaweed and cyanobiont.

**Supplementary Information:**

The online version contains supplementary material available at 10.1007/s10482-021-01672-x.

## Introduction

Since the inception of the holobiosis concept by Meyer-Abich (1934) and the term ‘holobiont’ was coined by Margulis (1990), our view of multicellular organisms has changed. The notion that multicellular organisms are colonised by complex communities of microbes that affect their physiology and ecology, has given rise to new questions. Using amplicon or metagenome sequencing, large amounts of data have been obtained to characterise substrate and host associated microbial communities. While these technologies have facilitated a revolution in the field of microbial ecology, at the same time they have revealed that the extent of microbial diversity that has not been described and/or cultured is far greater than previously thought (Amann and Rosselló-Móra 2016). To study host-microbe interactions and microbial communities in general, it is important that more of these taxa are characterised. More isolates, new taxonomic descriptions and epitypifications are needed to achieve this and to ultimately upgrade the available reference records (e.g., SILVA; Quast et al. 2013, RefSeq; O’Leary et al. 2016) on which amplicon and metagenome sequencing approaches rely. However, while these culture-independent studies are on the one hand limited by the substantial number of unknown reads, they can at the same time help to point in which direction particularly relevant and undescribed species may be found.

In the course of a global study on the invasive seaweed *Agarophyton vermiculophyllum* (Ohmi) Gurgel et al. (synonym: *Gracilaria vermiculophylla*), using 16S rRNA gene amplicon sequencing, an operational taxonomic unit (OTU) classified to the cyanobacteria was detected as core holobiont member, i.e. in virtually every sampled host (Bonthond et al. 2020). Besides that the cyanobacterial OTU was associated with *A. vermiculophyllum* across its global distribution range, it was also the overall most abundant OTU in the macroalgal holobiont and was rarely detected in the surrounding seawater. Based on comparison with the SILVA 16S rRNA gene database (Quast et al. 2013) the OTU was initially classified to the genus *Pleurocapsa* (Pleurocapsales). However, it only retrieved poor sequence hits using BLAST, which suggested it to be rather an undescribed related species or a relative without available sequence data. Cultivation efforts made for the present study yielded a non-axenic culture of a slow growing baeocytous cyanobacterium with some striking morphological differences to the genera *Pleurocapsa, Chroococcidiopsis* and other genera in the Pleurocapsales. The Pleurocapsales is an ecologically diverse group, including marine, freshwater, endolithic, epiphytic and sponge associated species (Al‐Thukair and Golubic 1991; Anagnostidis and Pantazidou 1991; Rippka et al. 2015; Konstantinou et al. 2018) that counts 247 species in 25 genera (Shalygin et al. 2019b and references therein). Many morphologically described species currently lack DNA sequence data of type material and it is suspected that the currently described taxa constitute only a minor portion of the actual species diversity residing in the order (Shalygin et al. 2019a).

The aim of the present work was to isolate, describe and sequence the genome of the pleurocapsalean cyanobacterium that clustered in Bonthond et al. (2020) into the core OTU associated with *A. vermiculophyllum*, to gain insight to its putative functional roles in the seaweed holobiont. Consequently, this study introduces *Waterburya agarophytonicola* gen. and sp. nov. based on the type strain *Waterburya agarophytonicola* KI4^**T**^. In addition, we highlight some genome characteristics that may be of relevance to the symbiosis with the host *A. vermiculophyllum* and based on this posit that the cyanobiont may represent an important source of cobalamin (vitamin B_12_) for its cobalamin auxotroph host.

## Methods

### Collection

During August/September 2017, algae of the Rhodophyte species *Agarophyton vermiculophyllum* (Fig. S1) were collected from several populations across the northern hemisphere (see Bonthond et al. 2020 for location details). One of the visited populations is located at the Falckensteiner Strand near the city Kiel (Germany, 54°23′55.3′′ N, 10°11′27.6′′ E). Aiming to either obtain axenic cultures of the host *A. vermiculophyllum* itself or cultures from intimately associated symbionts, we took samples of the youngest part of the algae (which are the apical tips) of approximately one millimetre length. To remove as many microbes as possible without harming the host, the tips were thoroughly rinsed with sterile artificial seawater (ASW) and transferred to 20 mL test tubes with aluminium caps containing 10 mL fresh sterile ASW and incubated at 15 degrees in near-darkness to create conditions where microbial productivity and proliferation would be minimal but the host is still able to grow. Over the period of approximately a year, the water was replaced a few times with fresh sterile ASW and the apical fragments rinsed with fresh sterile ASW as well. During a visual inspection in late 2018, one of the incubations contained high numbers of cyanobacteria-like cells that appeared to express baeocytous growth. A small number of the cells was transferred to a new vial and this subculture (without host) was incubated under the same conditions. While we were successful maintaining this subculture (labelled KI4), microscopic examination showed that it still contained other (but much smaller) bacterial cells and attempts to obtain completely axenic cyanobacterial isolates from KI4 by further subculturing single cells failed as the cells either died or were still non-axenic. An herbarium specimen was prepared from a subsample of KI4 by filtration of the medium and cells through a 0.2 µm membrane. The filter containing the cyanobacterial cells was submitted to the Algal herbarium of Natural History Museum of Denmark, Copenhagen.

### DNA extraction and genome sequencing

Due to the limited amount of cells and the slow growth, a DNA extraction from strain KI4 did not resolve detectable DNA concentrations. Therefore, a PCR was conducted directly on cells from the culture. This was done with the universal forward primer 27F; 5′-AGAGTTTGATCMTGGCTCAG-3′ and a reverse primer specific for the Pleurocapsales OTU from Bonthond et al. (2020, pleuro592R; 5′-CACTGCTTGCCAGAAGTTG-3′). The product was sequenced in both directions at the Institute of Clinical Molecular Biology in Kiel using an Applied Biosystems 3730xl DNA Analyzer and the sequence was deposited in Genbank (accession: MW113706).

To sequence the genome, we also transferred one to a few cells from the culture to two PCR tubes and used the EquiPhi29 DNA Polymerase (Thermo Fisher Scientific) for whole genome amplification, following the protocol of the manufacturer with the incubation step at 45 °C for one hour. Both samples and a blank were submitted to the Beijing Genomics Institute (BGI, Shenzhen, China) where library preparation was conducted and both samples were sequenced on the MGISEQ-2000 platform.

### Processing of sequence data, genome analysis

As the culture of *Waterburya agarophytonicola* KI4^**T**^ was not axenic, the genome sequence data was treated as a metagenome and the assembly was performed using the tools that are available in the software wrapper METAWRAP v1.3.2 (Uritskiy et al. 2018). In brief, raw read files were trimmed using cutadapt v1.18 (Martin 2011) and assembled with SPAdes v3.13.0 (Bankevich et al. 2012) with the metaSPADES option (Nurk et al. 2017) and the default error correction tool and K-mer sizes of 21, 33 and 55. Initial bin predictions were done with CONCOCT v1.1.0 (Alneberg et al. 2014), MaxBin2 v2.2.6 (Wu et al. 2016) and metaBAT2 v2.12.1 (Kang et al. 2019). Bins were refined and evaluated with CheckM v1.0.12 (Parks et al. 2015), reassembled with SPAdes and classified with Megablast (Altschul et al. 1990). The reassembled bin corresponding with *Waterburya agarophytonicola* was extracted and annotated automatically with the prokaryotic genome annotation pipeline (PGAP) from NCBI (Tatusova et al. 2016) and the integrated microbial genomes annotations pipeline (IMGAP) v5.0.20 from the JGI-IMG portal (Chen et al. 2021). The sequence files were deposited in Genbank (accession: PRJNA680001) and IMG (accession: 246459). To identify secondary metabolite clusters we used ANTISMASH v5.2.0 (Gruene et al. 2018) with default settings.

### Abundance of *W. agarophytonicola* on the host

To compare occurrence of the *W. agarophytonicola* core OTU from Bonthond et al. (2020) among seawater, algal surface and algal tissue, OTU counts from populations for which seawater, algal surface and algal tissue were sampled, were extracted from the 16S-V4 rRNA gene read count table of the respective study. To compare read counts in proportions of the total count among sampled substrates (seawater, algal surface, algal tissue), a generalised linear mixed model was fitted, assuming a beta binomial distribution and a logit in the link function (Douma and Weedon 2019) and with population and individual identity as random intercepts, using the R package glmmTMB (Magnusson et al. 2017, sampling and population details in Bonthond et al. 2020).

### Phylogenetic analyses

A total of 9593 sequences (16S rRNA genes, with average length about 1400 bp), including the majority of newly described cyanobacterial taxa and a full length 16S rRNA gene consensus sequence (1471 nucleotides) of *W. agarophytonicola* extracted from the initial genome assembly*,* were aligned in SINA (Pruesse et al. 2012) based on the secondary structure of the 16S rRNA molecule. A maximum likelihood analysis was conducted with FastTreeMP (Price et al. 2010) using the GTR + G substitution model with default settings and a 1000 bootstrap iterations, running on XSEDE (Towns et al. 2014) of the CIPRES Gateway (Miller et al. 2015).

In addition, 95 cyanobacterial genomes and the genomes of four outgroup taxa were downloaded to construct an alignment, including 31 conserved proteins (see Wu and Eisen 2008) which have been used in studies on cyanobacterial phylogenetics (Shih et al. 2013; Komárek et al. 2014; Österholm et al. 2020). With a few exceptions, the genomes used in these previous studies were also included in our analysis, which was, however, expanded with all currently available Pleurocapsales genomes in RefSeq (O’Leary et al. 2016) and the metagenome sequence of *Pleurocapsa mino*r HA4230-MV1^**T**^, which was recently designated as epitype for *P. minor* (Shalygin et al. 2019b) and sequenced (Ward et al. 2021). Genomes from *Rhodobacter sphaeroides* 2.4.1, *Chlorobium tepidum* TLS, *Chloroflexus aurantiacus* J-10-fl and *Heliobacterium modesticaldum* Ice1 were acquired to serve as outgroups in the analysis. Using the 31 protein sequences from the *Stanieria cyanosphaera* PCC 7437^** T**^ as query, a local *tblastn* (Camacho et al. 2009) was conducted against all downloaded genomes and the draft of *W. agarophytonicola* KI4^**T**^. Amino acid sequences of the best hits from each genome were aligned by protein using MAFFT v7.475 (Katoh et al. 2002). Alignments were then examined, trimmed and concatenated manually, resulting in a 9245 amino acid alignment. A Maximum-likelihood phylogenetic analysis was conducted with RAxML-HPC2 v8.2.12 (Stamatakis 2014) on XSEDE (Towns et al. 2014) using the PROTGAMMA model and partitioned protein substitution models, selected based on the Akaike information criterion, and a 1000 bootstrap iterations.

## Results and discussion

### Relative abundance in the holobiont

**Fig. 1:**
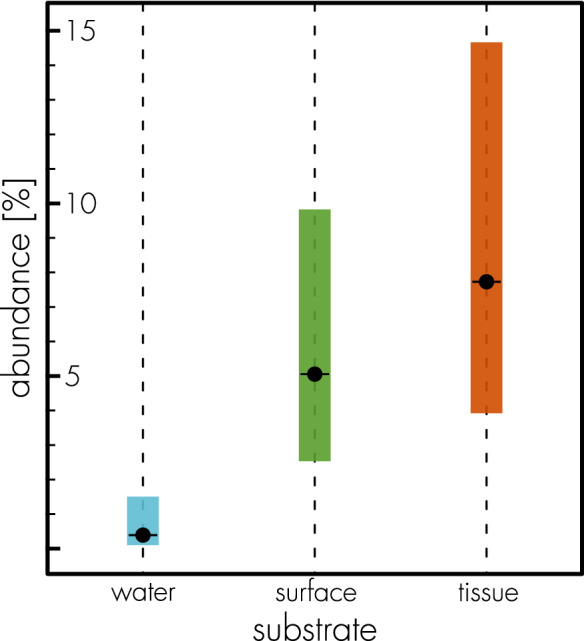
Estimated abundances of the *W. agarophytonicola* core OTU, across sampled substrates (i.e., seawater, algal surface and algal tissue) from 6 populations (Japan, China, Germany, France, Virginia, California, details in Bonthond et al. 2020). The 95% confidence intervals are indicated with shaded columns. Estimates and intervals were back-transformed from the log scale and multiplied by a 100 to be presented in percentages

The most abundant OTU from Bonthond et al. 2020, which closely related to *W. agarophytonicola*, constitutes on average 7.37% of all amplicon reads in tissue samples but also represents 4.68% of the reads in samples taken from the algal surface. However, in the seawater surrounding the holobiont the OTU is with a 0.39% average read count rare (Fig. 1). These occurrence data show that *W. agarophitonicola* is specifically found on *A. vermiculophyllum* and suggest it may occur as both endo- and epiphyte on its host.

### Phylogenetic analyses

**Fig. 2:**
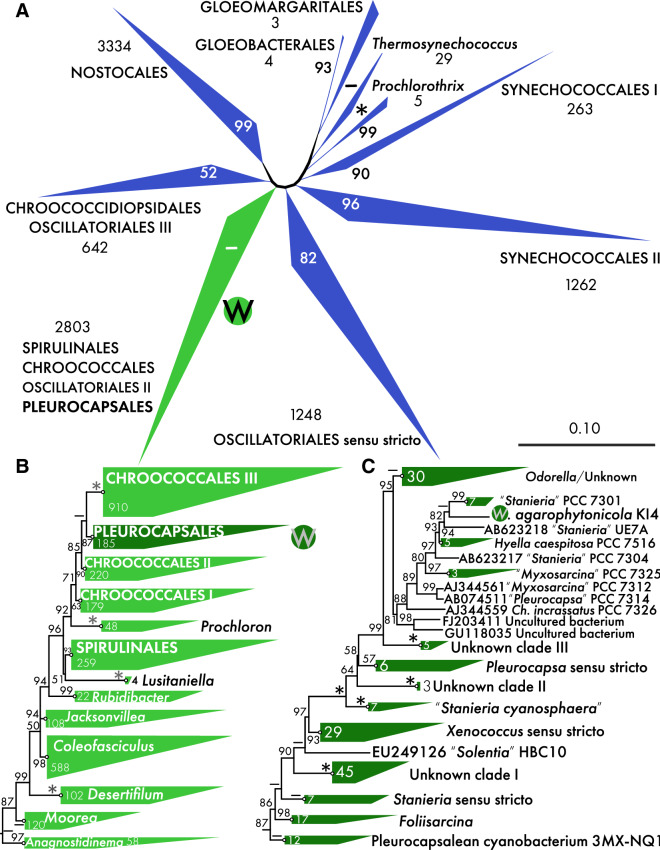
16S rRNA megaphylogeny with a total of 9593 taxa, showing the position of the new genus and species *Waterburya agarophytonicola*. **a** General view on the collapsed phylogeny with leaves depicting major cyanobacterial orders. Units near the names of the orders show the number of sequences in the collapsed clades. High support values on the backbone are not shown (they were 89–99); the node connecting the *Prochlorothrix* clade with the rest of the phylogeny did not show high support. **b** Zoomed view on the clade containing the order Pleurocapsales, with several Chrooccocales clades as sister taxa. **c** Detailed view of the order Pleurocapsales, including *W*. *agarophytonicola*. A large asterisk indicates the maximum support value of the Maximum Likelihood, “hyphen” depicts support < 50. *Note* **a**, **b** and **c** are the same tree with different levels of resolution (**b** and **c** are zoomed parts of the tree focused on Pleurocapsales and on *Waterburya agarophytonicola*). See Fig. S2 for an uncollapsed version of the order Pleurocapsales fraction in panel **c**

**Fig. 3:**
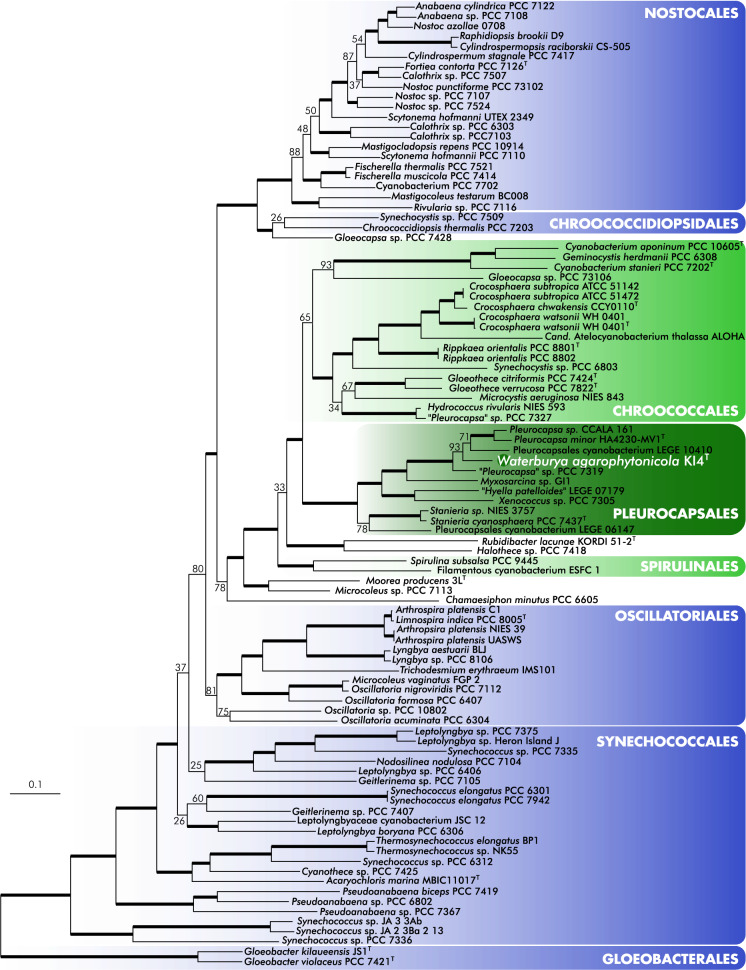
Maximum-likelihood tree based on 31 conserved proteins extracted from 96 cyanobacterial genomes. The analysis was conducted with 4 outgroup taxa which have been removed from the figure. *Waterburya agarophytonicola* is displayed in bold. Branches corresponding to nodes with > 95 bootstrap support values are thickened and ex-type strains are labelled with an uppercase ‘T’

The 16S rRNA gene phylogeny showed a typical clustering of cyanobacterial clades at the order level (Fig. 2a, Zimba et al. 2021). Only Nostocales and Gloeobacterales were monophyletic. The order Pleurocapsales with the new taxon *Waterburya agarophytonicola* was found within a large clade (2803 sequences) containing Chroococcales, Oscillatoriales and Spirulinales. A well supported clade (87% on Fast Maximum Likelihood) including 185 pleurocapsalean sequences was in the neighbouring position to the paraphyletic Chroococcales (Fig. 2b). A large clade including *W. agarophytonicola* and, surprisingly, the pseudofilamentous *Hyella caespitosa* PCC 7516 together with the nanocyte producer *Chamaecalyx incrassatus* PCC 7326 were in the sister position to the newly described *Odorella benthonica* clade (Fig. 2c). The cryptic clade of *Stanieria *sensu stricto was relatively distant from *W. agarophytonicola* and the new taxon *W. agarophytonicola* definitely forms an independent lineage. Whether or not the neighbouring sequences are part of the new genus *Waterburya* is a question outside of this study, for which detailed p-distance analysis and analyses of 16S-23S ITS rRNA in all of the respective neighbouring strains would be required. It can, however, already be stated that “*Stanieria*” PCC7103 and “*Stanieria*” UE7A have been previously misidentified as members of *Stanieria* and are either members of *Waterburya* or belong to separate genera (similarity values among these taxa and *W. agarophytonicola* are around 97%). The clade distribution in the multiprotein phylogeny (Fig. 3) is similar to phylogenies from previous studies based on the same set of proteins (Shih et al. 2013; Komárek et al. 2014; Mareš 2018; Österholm et al. 2020). Furthermore, the phylogenetic position of *W. agarophytonicola* in the multiprotein tree is in line with our 16S rRNA gene analysis (sister to *Pleurocapsa* sensu stricto). In the multiprotein tree, *W. agarophytonicola* grouped adjacent to the clade containing the recently epitypified *Pleurocapsa minor* HA4230-MV1 (Shalygin et al. 2019b). The same study also selected a neotype for the generic type; *Pleurocapsa fuliginosa*, which is closely related to *P. minor* (both grouped in the 16S rRNA gene phylogeny under *Pleurocapsa *sensu stricto, see Fig. 2. and Fig. S2). Besides the clear distinct morphology (e.g., *P. minor* and *P. fuliginosa* both form pseudofilaments), as a macroalgal symbiont, *W. agarophytonicola* is also ecologically unique and differs from the *Pleurocapsa* species which were both isolated from rocky substrates (Shalygin et al. 2019b). However, future study is needed to elucidate whether *W. agarophytonicola* can be found on other substrates and if the genus may accommodate more species with similar symbiotic lifestyles.

### General genome statistics

The draft of *Waterburya agarophytonicola* KI4^**T**^ counts 5,107,674 bp and is predicted to be 98.83% complete, consisting of 149 scaffolds with an average 289 × coverage and an N50 of 51,633. This is similar to a recent study from (Brito et al. 2020) who published a short-read based genome assembly of the related *Hyella patelloides* LEGE 07179 counting 675 scaffolds, and may indicate a high proportion of repetitive regions. With a predicted number 5,168,141 bp in total and an estimated gene count of 4902, the genome of *W. agarophytonicola* is the smallest of all 9 genomes currently available in the Pleurocapsales, of which *Pleurocapsa* sp. CCALA 161 has the next smallest genome (counting 5,463,308 bp and 5033 genes) and *Pleurocapsa* sp. PCC 7319 has the largest (7,386,997 bp, 6749 genes). The draft includes 36 tRNA genes, 3 noncoding RNAs, a transfer-messenger RNA gene and 4842 protein coding genes. As the assembly is based on short reads only, the number and order of ribosomal operon copies could not be resolved. The reassembled genome does not contain 16S rRNA gene copies and the full 16S rRNA gene sequence used for the 16S phylogeny (Genbank accession OK044280) was retrieved from the initial assembly and likely represents a consensus sequences of the different copies. Functions were predicted for 3375 genes, resulting in a total count of 1467 hypothetical proteins (Table 1). Besides *W. agarophytonicola*, the metagenome assembly of the culture contained 5 additional bins that were predicted to be > 70% complete, which were classified to the Proteobacteria (i.e., Alteromonadaceae, Methylophilaceae, Alphaproteobacteria, Gammaproteobacteria) and Bacteriodetes (Flavobacteriaceae, see Table S1). In line with the absence of cyanobacterial bins in the assembly, other cyanobacteria were never observed microscopically from KI4, supporting that the non-axenic culture is at least uni-cyanobacterial.

**Table 1:** Attributes of the *Waterburya agarophytonicola* KI4^T^ draft genome

Attribute	bp	Genes	% of total
Genome size	5,107,674		100.00%
DNA coding	4,436,783		86.87%
GC content	1,978,698		38.74%
DNA scaffolds	149		100.00%
Total gene count		4902	100.00%
RNA genes		40	0.82%
tRNA genes		36	0.73%
Regulatory and miscellaneous features		20	0.41%
Protein coding genes		4842	98.78%
With function prediction		3375	68.85%
With enzymes		922	18.81%
Connected to KEGG pathways		1046	21.34%
Connected to KEGG Orthology		1730	35.29%
Connected to MetaCyc pathways		798	16.28%
With COGs		3270	66.71%
With Pfam		3551	72.44%
With TIGRfam		1145	23.36%
With SMART		1084	22.11%
With SUPERFam		3757	76.64%
With CATH FunFam		2966	60.51%
In internal clusters		1156	23.58%
Coding signal peptides		170	3.47%
Coding transmembrane proteins		1163	23.73%
COG clusters		1658	50.70%
Pfam clusters		2030	57.17%
TIGRfam clusters		893	77.99%

The *W. agarophytonicola* genome contains six KEGG-classified genes that are absent in all other currently available Pleurocapsales genomes (Table S2). These are p-hydroxybenzoate monooxygenase, hydroxymethyl cephem carbamoyltransferase, L-ectoine synthase, 7,8-dihydropterin-6-yl-methyl-4-(beta-D-ribofuranosyl)aminobenzene 5′-phosphate synthase, a solute carrier family 10 (sodium/bile acid cotransporter) protein and an uncharacterised protein possibly involved in the biosynthesis of archaeosine. Altogether 73 COG-classified genes that are present in the *W. agarophytonicola* genome are absent from other known genomes of Pleurocapsales (Table S3).

### Metabolic features

Besides genes encoding proteins involved in pathways for photosynthesis and carbon fixation, *W. agarophytonicola*’s draft contains a duplicated set of the cytochrome c oxidase subunits I-III and the genes for succinate dehydrogenase/fumarate reductase (*sdhA, -B, -C*), which may indicate the capacity for a heterotrophic lifestyle as well.

While the capacity to fix nitrogen is common in the Pleurocapsales (Rippka et al. 2015) and the nitrogenase proteins are present in several of the sequenced Pleurocapsales genomes (i.e., *Myxosarcina* sp. GI1, Pleurocapsales sp. LEGE 06147, Pleurocapsales sp. LEGE 10410 and *Xenococcus* sp. PCC 7305, Table S1), none of the *NifD*, -*H* or -*K* genes were found in *W. agarophytonicola* KI4^**T**^. Therefore, the genome does not support a nitrogen fixing role for *W. agarophytonicola* in the *A. vermiculophyllum* holobiont, as was hypothesised in Bonthond et al. (2020, 2021). However, the draft contains the ferredoxin-nitrate and ferredoxin-nitrite reductase (*NarA*, *NirA*), and also reveals several transporter genes for extracellular nitrate/nitrite (*NrtA, -B, -C*). Therefore, the abundant occurrence of *W. agarophytonicola* may nonetheless affect nitrogen fluxes in the *A. vermiculophyllum* holobiont at the microscale, decreasing nitrate and nitrate concentrations in exchange for ammonia.

Further, a genetic basis for assimilatory sulfate reduction is present and this pathway is linked with genes catalysing sulfite production from Alkanesulfonate, 3'-phosphoadenosine-5'-phosphosulfate (PAPS), adenosine-5'-phosphosulfate (PAPS), methanesulfonate and thiosulfate. *Waterburya agarophytonicola* KI4^**T**^ has a high number of genes coding for carbonic anhydrase (5 KEGG and 8 COG annotations), which is more than any of the other 8 Pleurocapsales genomes in the IMG database. In addition, *W. agarophytonicola* contains the genes coding for phycobilisome proteins, i.e.; allophycocyanin, phycocyanin, phycoerythrocyanin and phycoerythrin. Also several carotenoid biosynthesis pathways were found, suggesting *W. agarophytonicola* is capable of synthesising β-carotene, zeaxanthin and canthaxathin. Similar with several other Pleurocapsales the *W. agarophytonicola* genome further features both endoglucanase and β-glucosidase, as well as an endo-1,4-beta-xylanase (KEGG annotations, Table S1). Besides these enzymes for cellulose and hemicellulose degradation the genome contains four copies of catechol 2,3-dioxygenase-like lactoylglutathione lyase (COG annotation; Table S2) that could enable *W. agarophytonicola* to degrade lignine. However, despite its apparent close and, based on the absence of records from other hosts or habitats, specific association with a red algal agarophyte, *W. agarophytonicola* features no agarase.

### Chemotaxis and adhesion

We found various chemotaxis and motility associated genes in *Waterburya agarophytonicola*’s draft genome, some of which in high copy numbers, which suggest the cyanobacterium may be responsive to different stimuli and be capable of directed movement. Notably, many KEGG clusters associated with chemotaxis are present, including genes for the methyl-accepting chemotaxis protein (MCP), purine-binding chemotaxis proteins (cheW) and the two-component system (cheA, -B, and -R). The genome also contains multiple copies of the chemosensory pili system protein ChpA and the twitching motility proteins (PilG, -H, -I, -J), as well as gene homologs required for positive phototactic motility (PixG, -H, -I, -J, -L). The genome further yields proteins associated with surface attachment and plant-microbe symbiosis, with COG annotations for18 genes coding cheY-like chemotaxis proteins, 3 genes coding signal transduction histidine kinase/cheY-like chemotaxis proteins and one gene for a two-component sensor histidine kinase/cheY-like chemotaxis protein. Moreover, the presence of 23 genes coding for filamentous hemagglutinin family (FHA) proteins and 5 copies for the large exoprotein involved in heme utilization and adhesion suggest *W. agarophytonicola* is well equipped with cellular mechanisms related to adhesion (Locht et al. 1993; Paulsrud and Lindblad 2002). Hemagglutinins such as FHA proteins have been associated in adhesion, adherence and virulence in plant pathogens (Gottig et al. 2009) but are also utilised by endophytic plant mutualists (Taghavi et al. 2010).

In addition, 8 genes coding for type IV pili (Tfp) assembly proteins (Pil) and 2 genes coding for uncharacterised surface proteins with fasciclin (FAS1) repeats, may further support the presence of adhesion and/or infection mechanisms. Such pili are, for example, found in plant endophytic *Nostoc* cyanobacteria, where they are expressed abundantly on the surface of hormogonia, and allow gliding motility towards the plant host (Duggan et al. 2007; Adams and Duggan 2012). Tfps are typical for a plant endophytic lifestyle (Frank 2018) and have also been found in unicellular and baeocytous cyanobacteria (Herdman and Rippka 1988) and even in bacteria from other phyla, where they may function in motility, adhesion, DNA exchange and pathogenesis (Mattick 2002; Adams and Duggan 2012).

### Vitamins

Similar to the other available Pleurocapsales genomes *W. agarophytonicola* KI4^**T**^ has the genetic basis for the synthesis of various vitamins, including biotin (B_7_), folate (B_11_), nicotinic acid (B_3_), panthothenate (B_5_), riboflavin (B_2_), thiamine (B_1_), α-tocopherol (E), phylloquinone (K_1_), menaquinone (K_2_) and pyridoxal 5’-phosphate (B_6_). Moreover, the genome contains the pathway for the synthesis of cobalamin (vitamin B_12_), including genes encoding cob(I)alamin adenosyltransferase and adenosylcobinamide-GDP ribazoletransferase. As the production of vitamins is energetically expensive, these metabolites may represent potential benefits to *W. agarophytonicola*’s host. In particular, vitamin B_12_ (cobalamin) is likely a valuable molecule for the red algal host. Most Rhodophyta, including the Florideophyceae –to which *A. vermiculophyllum* belongs– express the cobalamin-dependent methionine synthase (METH, Provasoli and Carlucci 1974) but are cobalamin auxotroph and depend on microbial symbionts to acquire this vitamin (Croft et al. 2005). Cobalamin auxotrophs may obtain the vitamin in exchange for fixed carbon (e.g., glycerol, Kazamia et al. 2012). Also *Gracilariopsis chorda*, the closest relative of *A. vermiculophyllum* of which a genome sequence is currently available, encodes METH and is thus dependent on the acquisition of cobalamin from microbial sources. Given the core and dominant presence of *W. agarophytonicola* in the holobiont (Bonthond et al. 2020), this is an important observation and could imply that the cyanobacterium represents a major or primary cobalamin source for *A. vermiculophyllum*. If this is true, and cobalamin from *W. agarophytonicola* is indeed functioning as a coenzyme in *A. vermiculophyllum* METH, another important question is what the cyanobacterium receives from the host in return. Rhodophytes produce various carbohydrates (Ito and Hori 1989) and as *W. agarophytonicola* has the genetic basis for aerobic respiration, one hypothesis could be that the cyanobiont is able to switch to a heterotrophic lifestyle, utilising both oxygen and a carbon source such as glycerol from the host. This would also avoid the necessity to compete for light between the two phototrophs.

### Secondary metabolites and siderophores

In total 15 secondary metabolite clusters were detected, including 6 non-ribosomal peptide synthetases (NRPSs) or NRPS-like gene clusters, 3 terpenes, 2 bacteriocins, 1 lanthidin, 1 ectoine, a T3-polykethinde synthase (PKS) cluster and a hybrid NRPS-T1PKS cluster. Some of these clusters may indicate the capacity for toxin production, including e.g., bacteriocins, which are toxins often produced to inhibit growth of other bacteria (Cotter et al. 2013) and NRPSs, PKSs or hybrids that may be involved in the synthesis of various toxins, including, Anatoxin-A, Cylindrospermopsin and microcystin (Kehr et al. 2011).

NRPSs are also commonly at the basis for the synthesis of siderophores and other metallophores (Årstøl and Hohmann-Marriott 2019; Kramer et al. 2020). The genome also contains a copy of the menaquinone-specific isochorismate synthase, a key enzyme in the synthesis of a siderophore group containing NRPSs (Walsh and Gary Marshall 2004). Iron or other metal scavenging compounds are produced by most bacteria and operate extracellularly. Siderophores may increase the local iron availability not only to their producer but at the same time to other microbes with the matching receptors for uptake. While their production can thus be favourable to selective taxa, they may at the same time promote iron starvation in other taxa and therewith strongly influence the taxonomic composition in microbial communities (Kramer et al. 2020). Mutualistic associations between terrestrial plants and siderophore producing endophytic bacteria have been documented for some time (Loaces et al. 2011; Frank 2018) and also for macroalgae it is thought that microbial mutualists play an important role in the regulation of availability of iron and other trace metals (Wichard 2016). The genome further reveals three genes coding for ferric uptake regulators (Fur), a common key regulator in synthesis and activity of siderophores (Kramer et al. 2020) and several other proteins related to siderophore transport, including 9 major facilitator superfamily (MFS) permeases and TonB, ExbB, ExbD transporter proteins (Årstøl and Hohmann-Marriott 2019, Tables S1, S2). Altogether, the presence of these different genetic signatures associated with siderophore synthesis thus hint at a role for *W. agarophytonicola* in the cycling of iron and/or other trace metals within the holobiont.

### Taxonomy

The genus *Waterburya* and the species *W. agarophytonicola* are here described according to the International Code of Nomenclature for algae, fungi, and plants (Turland et al. 2018).

## *Waterburya* Bonthond and Shalygin gen. nov.

### Diagnosis

Akin to *Stanieria* by morphology, different from which by marine habitat, epi-endophytic growth on the rhodophyte *A. vermiculophyllum*, and based on 16S rRNA gene phylogeny.

### Description

In culture, small groups of cells forming blackish visible aggregates (macrocolonies). Microcolonies consist of mostly individual, spherical cells; or oblate-spherical cells in groups growing together. Cells in the microcolony of different sizes, terracotta or pale grey-brown by color. Cells surrounded by thin, colorless mucilaginous envelopes, never with common slime. Baeocytes formation through multiple fission of the mature cells, which are much larger than baeocytes. Baeocytes are released by cell wall breakage. Reproduction exclusively by baeocyte production.

### Etymology

Named in honour of John B. Waterbury—an important cyanobacterial researcher, who worked on morphology and physiology of many marine baeocyte producers.

### Type species

*Waterburya agarophytonicola*

## *Waterburya agarophytonicola* Bonthond and Shalygin sp. nov. (Fig. 4)

**Fig. 4:**
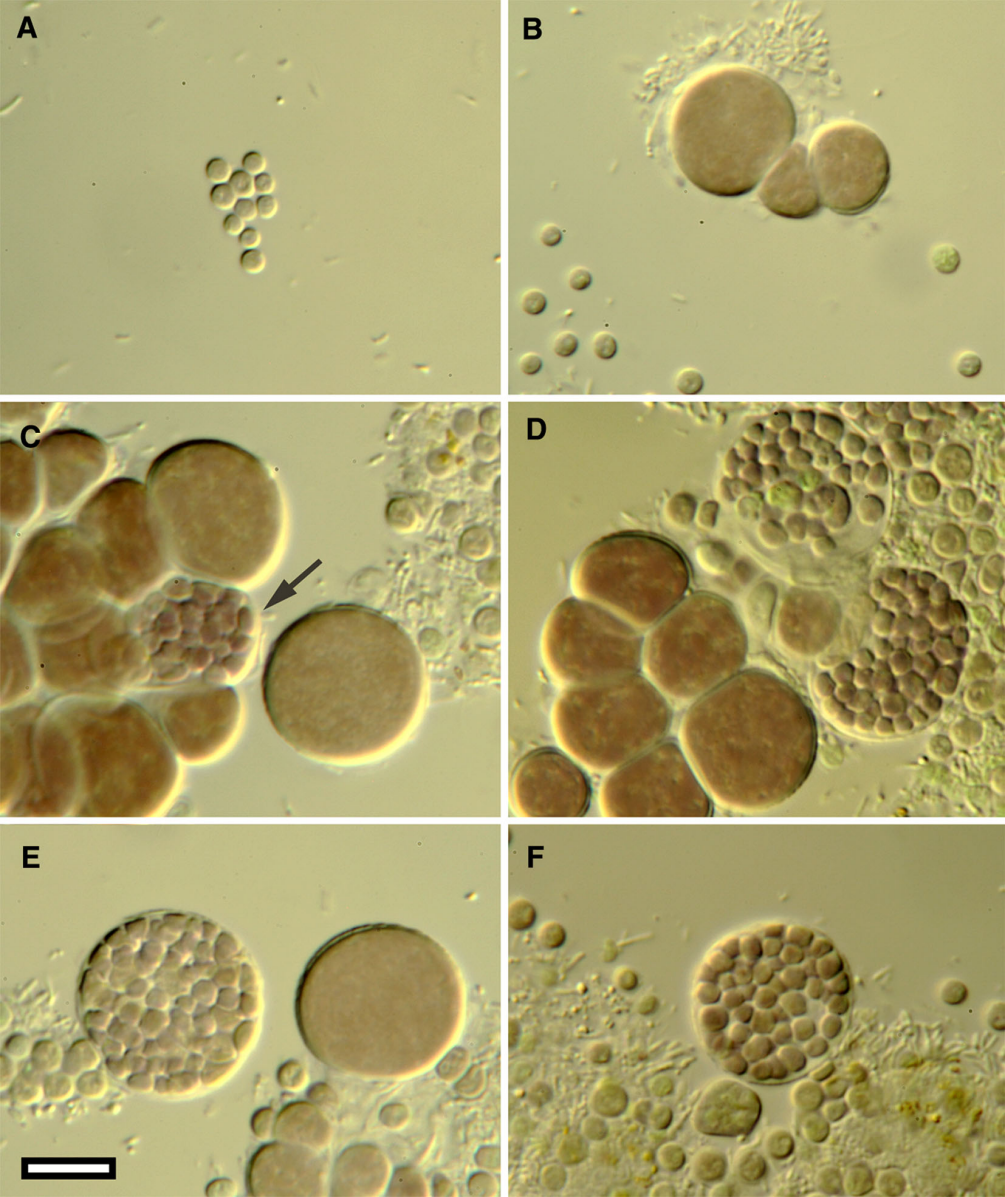
Microphotographs showing the morphology of *Waterburya agarophytonicola*. **a** Released baeocytes; **b** Growing cells, note that adjacent cells are not a product of binary fission; **c** Aggregation of the cells and initial baeocyte formation within mother cell (arrow); **d** Massive baeocyte formation adjacent to the small group of growing cells; **e** Clear example of baeocyte production; **f**: Unreleased, released and growing baeocytes. The scale bar equals 10 μm

### Holotype

Here designated: Specimen on filter, lyophilised. Algal herbarium of Natural History Museum of Denmark, Copenhagen; accession number: C-A-99685.

### Diagnosis

Morphologically similar to *Stanieria sublitoralis*, but differing by phylogenetic position on the 16S rRNA gene tree and by being associated with *Agarophyton vermiculophyllum*. Akin to *Chroococcopsis gigantea*, different by 16S rRNA gene phylogeny, smaller cells, lacking daughter cells and its distribution in marine to brackish habitats.

### Description

Macrocolonies slowly growing in liquid BG11 medium (20 PSU) under low light conditions in form of tight blackish clusters. Microcolonies small, consisting of released baeocytes (3.5–6 μm in diameter), growing baeocytes (up to 8 μm in diameter), and large, mature cells ready to form baeocytes through multiple fission (7–20 μm in diameter). Mucilaginous envelopes colorless, adherent to the cell walls, rarely slightly widened and more robust especially in the stressed cultures. Released baeocytes growing to large sizes (up to 19–20 μm), after which multiple fission occurs. Sometimes baeocytes form in the smaller mother cells, 10–15 μm. Growing cells spherical or oblate-spherical, terracotta by color, released baeocytes pale grey-brown. Unreleased baeocytes abundant, 64 or 128 per one mother cell; small, 2–2.5(3) in diameter.

### Etymology

*Agarophyton-* from the marine red alga *Agarophyton vermiculophyllum* (Ohmi) Gurgel et al*.,* and *-cola* from L. n. *incola*; an inhabitant*.*

### Type locality

Falckensteiner Strand Kiel (54°23′55.3′′ N, 10°11′27.6′′E), within one meter from the surface (Baltic sea) on *Agarophyton vermiculophyllum*.

### GenBank accession numbers

MW113706 (16S rRNA gene partial sequence obtained with Sanger sequencing), OK044280 (16S rRNA gene complete, from initial genome assembly), PRJNA680001 (draft genome).

### Taxonomic notes

The genus *Stanieria* is highly polyphyletic, even though the type species *S*. *cyanosphaera* PCC 7437 was phylogenetically established. There are at least 5 phylogenetically distant clades resembling members of *Stanieria* outside of the *Stanieria* sensu stricto clade. One of them contains *Stanieria* sp. PCC 7302 isolated from a seawater tank at California Bay (Mexico) (Waterbury and Stanier 1978). This isolate is similar by morphology to *Waterburya agarophytonicola,* however different by habitat and 16S rRNA gene phylogeny. According to Anagnostidis and Pantazidou (1991), *Stanieria* sp. PCC 7302 may belong to the established taxon *Stanieria sublitoralis* (A. Lindstedt) Anagnostidis & Pantazidou, but this is questionable, considering vast differences in ecology and geography between these two taxa (isolated in a Swedish sublittoral on various sea animals, valves and algae versus isolated from a water tank in Mexico). To confirm the true affiliation of *Stanieria sublitoralis* DNA sequencing of type material from the type locality is required.

## Conclusion

With this work we introduce *Waterburya agarophytonicola* Bonthond and Shalygin gen. nov. sp. nov. and present a draft of its genome. While the exact nature of this rhodophyte-cyanobacterium symbiosis remains to be determined in future work, the genome reveals clues to its functional roles as a core member in the *A. vermiculophyllum* holobiont (Bonthond et al. 2020). Altogether, a high number of chemotaxis, adhesion and adherence related genes support a host-associated lifestyle for *W. agarophytonicola*. Genes for adherence and virulence (genes for Tfps and FHAs), combined with occurrence data (Fig. 1) especially hint at an endophytic lifestyle. However, we have not been able to microscopically confirm whether *W. agarophytonicola* occurs endo- and/or epiphytically and whether it is associated intra- and/or extracellularly, which thus remains a question for feature research. This study does not support a diazotrophic role for the cyanobacterium in the holobiont, but instead demonstrates it has the potential to function as a source of vitamins, in particular cobalamin, to the vitamin B_12_-auxotrophic host. In addition, *W. agarophytonicola* may possibly facilitate uptake of iron and/or other trace metals by its host. To further investigate the relationship between *W. agarophytonicola* and *A. vermiculophyllum*, advanced microscopic work is needed to confirm the endo- and/or epiphytic occurrence of the cyanobiont and identify whether *W. agarophytonicola* produces chlorophyll in association with the host, to decipher if it can adapt a heterotrophic lifestyle. Moreover, this may help to explore whether pili-like structures, such as those richly reflected in the genome, are expressed by the cyanobiont. To shed light on how specific the relationship between *A. vermiculophyllum* and *W. agarophytonicola* is, related hosts need to be studied as well. Finally, metabolic assays should be carried out in an experimental context to quantify cobalamin production and to screen for host cell wall carbohydrates or other host molecules that are potentially metabolised by *W. agarophytonicola*.

## Supplementary Information

Below is the link to the electronic supplementary material.

*Agarophyton vermiculophyllum* (dark red) fixed to hard substratum at the Cherrystone Campground in the Chesapeake Bay along the Eastern Shore of Virginia. ©SA Krueger-Hadfield (PDF 3687 kb)

An uncollapsed fraction of the Maximum likelihood 16S rRNA phylogeny of the Pleurocapsales. The displayed tree is the fraction of the phylogeny shown on the Fig. 2C. Branches corresponding to nodes with full bootstrap support are indicated with stars and nodes with < 50 support values are labelled with a dashes (PDF 234 kb)

Supplementary file3 (XLSX 288 kb)

## Data Availability

The holotype *Waterburya agarophytonicola* KI4^**T**^ was deposited in the Algal herbarium of Natural History Museum of Denmark, Copenhagen; accession number: C-A-99685. The partial 16S rRNA gene Sanger sequence and a full 16S rRNA gene consensus sequence obtained from the initial genome assembly were deposited in Genbank under the accessions MW113706 and OK044280, respectively. The draft genome assembly and PGAP annotation are available in the Short Read Archive (SRA, accession PRJNA680001). Annotations from the IMGAP can be accessed on IMG (Taxon ID: 2913235824, submission ID: 246459).
