# Supplementary figures and images for: Draft genome and description of Waterburya agarophytonicola gen. nov. sp. nov. (Pleurocapsales, Cyanobacteria): a seaweed symbiont
Source: Antonie Van Leeuwenhoek. 2021 Oct 21;114(12):2189–203. doi: 10.1007/s10482-021-01672-x (PMC8580901; doi:10.1007/s10482-021-01672-x)

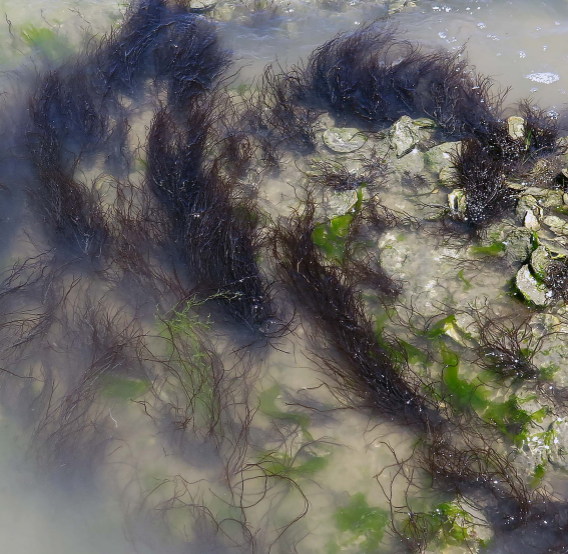

Supplement: Supplementary file 1 — Agarophyton vermiculophyllum (dark red) fixed to hard substratum at the Cherrystone Campground in the Chesapeake Bay along the Eastern Shore of Virginia. ©SA Krueger-Hadfield (PDF 3687 kb) [file 10482_2021_1672_MOESM1_ESM.pdf]

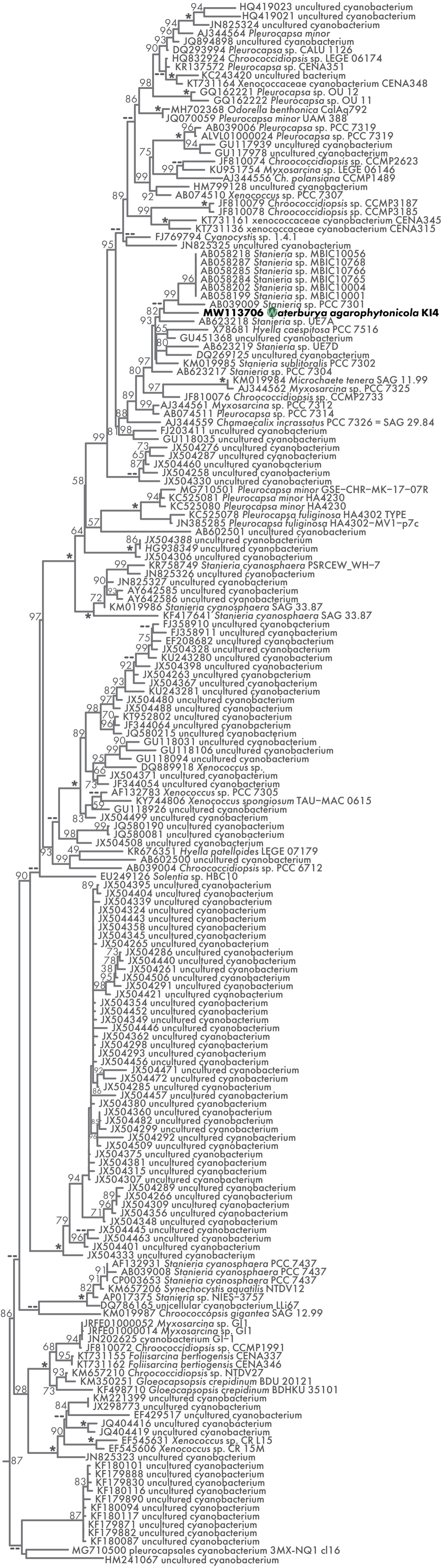

Supplement: Supplementary file 2 — An uncollapsed fraction of the Maximum likelihood 16S rRNA phylogeny of the Pleurocapsales. The displayed tree is the fraction of the phylogeny shown on the Fig. 2C. Branches corresponding to nodes with full bootstrap support are indicated with stars and nodes with < 50 support values are labelled with a dashes (PDF 234 kb) [file 10482_2021_1672_MOESM2_ESM.pdf]
